# The relationship between sleep quality and dyspnoea severity in patients with COPD

**DOI:** 10.4314/ahs.v20i4.32

**Published:** 2020-12

**Authors:** Emine Kaplan Serin, Emine Derya Ister, Ahmet Ozdemir

**Affiliations:** 1 Department of Nursing, The Faculty of Health Sciences, Gaziantep University, Gaziantep, Turkey; 2 Department of Nursing, The Faculty of Health Sciences, Kahramanmaraş Sütçü İmam University, Kahramanmaraş, Turkey

**Keywords:** Dyspnoea, COPD, Sleep

## Abstract

**Objectives:**

This study aimed to determine sleep quality, frequency and severity of dyspnoea in COPD patients and to evaluate the relationship between dyspnoea severity and sleep quality.

**Method:**

The sample of the study consisted of 110 patients admitted to the Chest Diseases polyclinic of a private hospital and diagnosed as COPD for at least one year. The data of the study were collected using the “Individual Information Form”, “COPD and Asthma Sleep Scale (CASIS)” and “Medical Research Council (MRC) Dyspnoea Scale”.

**Results:**

It was found that 6.4% of the patients did not experience dyspnoea, 34.5% had mild, 40.9% had moderate, and 18.2% had severe dyspnoea. The mean CASIS score of the patients without dyspnoea was 29.08±7.83, with mild dyspnoea was 40.22±9.30, with moderate dyspnoea was 50.31±8.97 and with severe dyspnoea was 56.96±13.13. There was a statistically significant difference between dyspnoea severity and mean CASIS score (p=0.001). Correlation analysis between MRC dyspnoea scale and CASIS score showed a significant positive correlation (r=0.61 p=0.001).

**Conclusion:**

It was concluded that the majority of COPD patients had moderate or poor sleep quality and dyspnoea. As dyspnoea severity increases, sleep quality decreases.

## Introduction

Chronic Obstructive Pulmonary Disease (COPD) is a progressive pulmonary disease characterized by irreversible airflow restriction, increased sputum production, and coughing, resulting in an abnormal inflammatory response of the lungs to certain particles and gases.^1^,^2^ Although COPD, which is one of the most important causes of death today, is largely preventable, it is difficult to treat after the onset of the disease and has a high financial burden.^1^,^2^ However, COPD patients experience many symptoms related to the disease. The most important and common symptoms of the patients are chronic and progressive dyspnoea, cough, sputum production and fatigue. Dyspnoea is the most critical symptom experienced by many COPD patients, characterized by air hunger, which lowers activity levels. Dyspnoea, which is the most common symptom in COPD, causes patients to experience intense fear, inadequacies in life activities, change in their priorities, increase in addictive states, fear of death, intense anxiety and depression. Dyspnoea also affects the sleep quality of the patients. Chang et al. (2016) reported that more than half of COPD patients had poor sleep quality and had problems waking up at night or early in the morning.^3^

Sleep quality of COPD patients is inferior in relation to respiratory problems.^5^,^6^ Disturbances in sleep patterns and respiratory changes due to the disease significantly affect the prognosis of the disease. Besides, insomnia caused by sputum production and cough is one of the common problems affecting daily living activities in COPD patients. Nocturnal symptoms and sleep disturbances are common in COPD in proportion to the severity of the disease.^7^ Sleep disorders seen in COPD occur in the form of insomnia, excessive sleep, changes in sleep patterns, and abnormal respiratory activity during sleep.^8^ COPD patients experience a range of disorders related to sleep and breathing, and respiratory sleep disorders associated with hypoxemia, resulting in reduced sleep quality.^9^

Sleep is one of the essential and fundamental requirements of human life. While changes in sleep order and quality affect daily life activities, prolongation of this change may cause deterioration in body and mental health. It is seen that patients with reduced sleep quality have difficulty in coping with the delaying and stressful situation during the healing process. Alteration of breathing during sleep includes physiological respiratory changes, which means pathological conditions, including deterioration in gas exchange.^10^ Although sleep disorders are common in COPD patients, these patients are vulnerable to increased gas exchange deviations wth sleep.^6^ While sleep oxygenation is insignificant in healthy adults, it may be severe and fatal in people with lung disease.^3^

## Aim

The primary aim of this study was to determine the frequency and severity of dyspnoea, and sleep quality in COPD patients; its secondary aim is to evaluate the relationship between sleep quality and severity of dyspnoea.

## Materials and methods

### Research design

This research was conducted in descriptive and relationship-determining design.

### Population-Sample

The sample of this study consisted of 110 patients who applied to the Chest Diseases Polyclinic of a private hospital between March 2017 and January 2018 and met the research criteria. The criteria for inclusion in the study were the diagnosis of COPD for at least one year and being 18 years of age or older.

### Data collection tools

In the collection of data, individual information form prepared in accordance with the literature, breathlessness scale (MRC) and asthma and COPD sleep scale (CASIS) were used.

**Individual Information Form:** This form consists of a total of 31 questions. The form includes questions about sociodemographic characteristics, disease and treatment, dyspnoea and sleep assessment.

**Medical Research Council (MRC) Dyspnoea Scale:** This scale was first used by Fletcher to compare the severity of dyspnoea during the activity of people with and without lung disease.^11^ Later, the British Medical Research Council introduced this scale in a more developed form in order to monitor the natural history of the disease.^12^ MRC is a five-item scale based on various physical activities that produce a feeling of dyspnoea. Here, patients are asked to mark the level of activity that causes dyspnoea in themselves.^13^

The items of MRC;

1. No Dyspnoea: No breathing difficulty when moving smoothly on a flat surface or a slight slope.
2. Mild Dyspnoea: Breathing difficulty when moving fast on a flat surface or a slight slope.
3. Moderate Dyspnoea: Walking slower than peers when walking on flat ground, pausing to breathe.
4. Severe Dyspnoea: Giving a breathing break after walking up to 100 meters or a few minutes
5. Very Severe Dyspnoea: Staying breathless while doing daily chores at home (wearing clothes, taking off or going to the toilet).

### COPD and Asthma Sleep Impact Scale (CASIS)

CASIS was developed by Pokrzywinski et al. to demonstrate the effect of asthma and COPD on sleep. The items of the questions on a scale of 7 questions in total are Likert in the form of never, rarely, sometimes, often, very often. The first five items are scored straight; the sixth and seventh items are scored upside down. Total raw points are obtained by collecting all item points. The scale score is calculated with the formula given below.

Scale Score = ×100

A high scale score indicates poor sleep quality and a low score indicates good sleep quality 14. The validity and reliability study of CASIS in Turkish was conducted by Ayhan and Kıyak, and CronbachAlfa was found to be 0.87.15 In this study, Cronbach Alpha value of CASIS was determined as 0.71.

### Data collection

Data were collected between March 2017 and January 2018.

### Analysis of data

SPSS 17 package program was used for data analysis. Descriptive (number, percentage, mean and standard deviation) and independent samples t-test, one-way ANOVA, Mann Whitney U test, Kruskal Wallis test and Pearson correlation analysis were used in the analysis of the data.

### Ethical aspects of research

This research was conducted in accordance with the principles of the Declaration of Helsinki. Approval was obtained from the hospital where the study was conducted and from the Non-Interventional Research Ethics Committee of Fırat University (2017/15). After obtaining the necessary legal permissions, verbal and written approvals were obtained by explaining the aims of the study to the patients who agreed to participate in the study.

## Results

It was determined that 54.5% of the patients were 61 years or older, 50.9% were women, 79.6% were married, 65.5% were literate, 79.1% were not working, and 86.4% had social security. 65.5% of the patients had a chronic non-COPD disease, only 17.3% had regular medical visits, 74.5% had been hospitalized at least once during the last year, 77.3% had used their medication regularly, 75.5% used nebulizer at home and 27.3% used oxygen tube at home. It was determined that 42.7% of the patients did not smoke, 29.1% continued to smoke, 28.2% quit smoking. 67.3% of the patients stated that they do not need any person to meet their care needs (Table 1).

**Table 1:** Some characteristics of patients

Variables	Number (n)	Percentage (%)
**Age**		
<60	50	45.5
61 and older	60	54.5

**Gender**		
Female	56	50.9
Male	54	49.1

**Marital status**		
Single	22	20.4
Married	86	79.6

**Education level**		
Illiterate	35	31.8
Literate	39	35.5
Primary school	16	14.5
Highschool	11	10.0
University	9	8.2

**Working status**		
Yes	23	20.9
No	87	79.1

**Financial situation**		
Income less than expense	18	16.4
Income equal to expense	52	47.3
Income higher than expense	40	36.4

**Social security**		
Yes	95	86.4
No	15	13.6

**Family structure**		
Nuclear family	90	81.8
Extended family	20	18.2

**Duration of diagnosis of COPD**		
1–5 years ago	51	46.4
6–10 years ago	27	24.5
11–18 years ago	32	29.1

**History of chronic disease other than COPD**		
Yes	71	64.5
No	39	35.5

**Smoking**		
Yes	32	29.1
No	47	42.7
Quit	31	28.2

**Getting education about the disease**		
Yes	73	66.4
No	37	33.6

**Going to regular doctor check-up**		
Yes	19	17.3
No	21	19.1
Partly	70	63.6

**History of hospitalisation in the last year**		
Never	28	25.5
1–2 times	37	33.6
3 and more	45	40.9

**Caregiver**		
Do not need care	74	67.3
Need care but no caregiver	8	7.3
Have a caregiver	28	25.5

**Using medication regularly**		
Yes	85	77.3
No	25	22.7

**Using a nebuliser at home**		
Yes	83	75.5
No	27	24.5

**Using an oxygen tube at home**		
Yes	30	27.3
No	80	72.7

It was determined that 93.6% of the patients had dyspnoea, and 34.5% of them had mild, 40.9% had moderate, and 18.2% had severe dyspnoea. Based on CASIS, 37.3% of the patients had poor sleep quality and 56.4% had moderate sleep quality. The rate of patients with good and very good sleep quality was determined as 6.3% (Table 2).

**Table 2:** Patients' dyspnoea severity based on MRC dyspnoea scale and sleep quality based on CASIS

	N	%
**Dyspnoea Level**		
No Dyspnoea	7	6.4
Mild Dyspnoea	38	34.5
Moderate Dyspnoea	45	40.9
Severe Dyspnoea	20	18.2

**Sleep Quality**		
Very good (less than 19 points)	3	2.7
Good (20–30 points)	4	3.6
Moderate sleep quality (31–49 points)	62	56.4
Poor sleep quality (40–75)	41	37.3

The comparison of the mean CASIS score of the patients with some variables is given in Table 3. Sleep quality was found to be lower in patients aged 61 years or older than patients aged 60 and under (p=0.001). There was no statistically significant difference between the mean CASIS scores of the patients according to gender, marital status, family structure, social security presence and smoking status (p>0.05). The difference between the mean CASIS scores according to the educational level of the patients was statistically significant (p=0.001). CASIS scores of high school and university graduates were lower than those of illiterate, literate and primary school graduates, and sleep quality was better.

**Table 3:** Comparison of mean CASIS score of patients with some variables

	CASIS mean±sd	Statistical Test p
**Age**		t=-4.51
<60	41.28±10.82	**p=0.001**
61 and older	51.19±10.95	

**Gender**		
Female	47.32±13.6	t=0.54
Male	46.03±11.13	p=0.589

**Marital status**		U=893.00
Married	46.88±11.15	p=0.684
Single	43.99±15.60	

**Family structure**		U=724.500
Nuclear	47.18±12.93	p=0.171
Extended	44.46±9.86	

**Education level**		
Illiterate	52.14±10.40	KW=22.299
Literate	48.07±10.77	**p=0.001**
Primary school	45.31±12.14	
Highschool	35.06±12.03	
University	36.11±13.53	

**Working status**		
Yes	39.13±10.07	U=508.00
No	48.68±12.27	**p=0.001**

**Social security**		
Yes	46.16±12.49	U=586.00
No	50.00±11.92	p=0.267

**Financial situation**		
Income less than expense	44.24±10.18	KW=6.09
Income equal to expense	49.24±14.09	**p=0.047**
Income higher than expense	44.46±10.51	

**MRC**		
No Dyspnoea	29.08±7.83	KW=41.534
Mild Dyspnoea	40.22±9.30	**p=0.001**
Moderate Dyspnoea	50.31±8.97	
Severe Dyspnoea	56.96±13.13	

**History of hospitalisation in the last year**		
Never	38.01±13.15	F=19.80
1–2 times	44.59±8.44	**p=0.001**
3 and more	53.80±10.72	

**Smoking history**		
Yes	43.75±13.57	F=1.50
No	47.11±13.04	p=0.226
Quit	49.07±9.75	

**History of chronic disease other than COPD**		
Yes	50.60±11.70	t=4.90
No	39.56±10.50	**p=0.001**

**Getting support for care**		KW=20.215
Yes	53.57±12.17	**p=0.001**
No (No need for care)	42.7±10.47	
No (Need care)	58.92±12.94	

The correlation analysis between the MRC dyspnoea scale and the CASIS score showed a significant positive correlation (r=0.61 p=0.001) (Table 4).

**Table 4:** The correlation analysis between the MRC dyspnoea scale and the CASIS

	CASIS	
MRC dyspnoea scale	r	p
Dyspnoea Level	0.61	**0.001**

## Discussion

In this study conducted to determine the sleep quality and dyspnoea status of COPD patients and to determine the relationship between dyspnoea and sleep quality, it was identified that 93.6% of the patients had dyspnoea. According to the MRC dyspnoea scale, 34.5% of patients had mild, 40.9% moderate and 18.2% had severe dyspnoea. In their study (2012), Yorgancioğlu et al. sampled 321 COPD patients and found that 78.2% of patients had dyspnoea complaints.^5^ Miravitlles et al. (2009) reported mild dyspnoea in 46.7%, moderate in 24.9%, and severe dyspnoea in 15.6% of COPD patients followed in primary care.^16^ The results of our study and the literature were found to be consistent.

In this study, it was found that 56.4% of the patients had moderate sleep quality, and 37.3% had poor sleep quality. Only 6.3% of patients have good and very good sleep quality. In a study conducted by Mohammad Ali Zohal et al. (2014) using Pittsburgh sleep quality index (PSQI) with 139 COPD patients in Iran, 74.8% of the patients were found to have poor sleep quality. In the same study, the rate of patients with good sleep quality was reported as 25.2%.^17^ In a study conducted by Akinci et al. (2017) with 51 moderate to severe COPD patients, it was reported that 94% of the patients with moderate and severe COPD patients had poor sleep quality measured by PSQI.^18^ In a study conducted by Steven M Scharf et al. (2011) in Israel, it was reported that 77.7% of the patients had poor sleep quality evaluated by PSQI.^19^ In the same study, it was reported that the sleep quality of the patients was related to the respiratory symptoms they experienced at night. In this study, only 6.4% of COPD patients do not have dyspnoea, whereas 93.6% experience mild to severe dyspnoea. In a study by Hynninen et al., it was reported that patients with increased COPD severity had more sleep problems, and night-time breathing problems, pain and psychological problems increased the complaints of insomnia.^20^ Omachi et al. reported that sleep disturbance was associated with cough symptoms, dyspnoea scale (breathlessness) and COPD severity in a study conducted with 98 COPD patients.^21^

In the present study, the mean CASIS score of patients 61 years and older was higher than that of patients younger than sixty years, and the difference between the two means was statistically significant (p=0.001). This finding shows that the sleep quality of COPD patients 61 years and older is worse. Kacaroğlu Vicdan (2018) reported that there was no difference between the sleep quality of 61 years and older patients and the sleep quality of 60 years and younger patients in a study conducted with 62 COPD patients using PSQI.^22^ In another study evaluating the sleep quality of COPD patients using PSQI, it was reported that sleep quality did not change according to age.^18^ This may be due to the assessment of sleep quality of patients with CASIS in our study.

In our study, no statistically significant difference was found between the gender, marital status, family structure, presence of social security, smoking history and mean CASIS score (p>0.05). Karacaoğlu Vicdan (2018) showed that there was no difference between the gender, marital status and educational level of the patients and their mean CASIS score.^22^ However, in this study, a statistically significant difference was found between the educational level of the patients and the mean CASIS score (p=0.001). Sleep quality of the patients with low education level was found to be low. High school and university graduates have higher sleep quality as assessed by CASIS.

There are numerous studies in the literature showing no correlation between smoking status and sleep quality in COPD patients.^7^,^18^,^23^ Similarly, in this study, the sleep quality of patients did not change according to smoking status. In the last year, a statistically significant difference was determined between hospitalization and non-COPD chronic disease history and mean CASIS score (p=0.001). In the study conducted by Dignani et al. (2016), the sleep quality of patients decreases as the stage of COPD increases. The same study reported a strong correlation between disease severity and sleep quality.^24^ In a study conducted by Hynninen et al., it was reported that patients with increased COPD severity had more sleep problems and more night-time breathing problems.^20^ Similarly, Omachi et al. reported that sleep disturbance in COPD patients is associated with COPD severity.^21^

According to the MRC dyspnoea scale, a statistically significant difference was found between dyspnea severity and mean CASIS score (p = 0.001). The average CASIS score of the patients without dyspnoea was lower than those with dyspnoea, and the higher the severity of dyspnoea, the higher the CASIS score. This finding shows that sleep quality decreases as the severity of dyspnoea increases. Also, according to the correlation analysis between MRC score and CASIS score, a strong positive correlation was found (r=0.61 p=0.001). Chang et al. (2016) found a positive correlation between MRC dyspnoea scale scores and poor sleep quality.^3^ Using actigraphy and PSQI, Nunes et al. (2013) found a statistically significant correlation between dyspnoea severity and sleep effectiveness (R=0.41), and total sleep duration (R=-0.46) in their study with COPD patients. In the same study, it was reported that MRC score is the best predictor of total sleep duration and sleep adequacy in COPD patients.^25^ Dignani et al. found a strong correlation between PSQI and MRC (r= .77). Omachi et al. reported that sleep disturbance is associated with dyspnoea severity in COPD patients.^21^ However, Akıncı et al. (2018) found no relationship between dyspnoea severity and sleep quality in COPD patients, while the authors stated that this might be due to the small sample size and low dyspnoea scores.^18^

## Conclusion

In this research, the following conclusions were reached.

- Sleep quality of patients is generally low,
- The vast majority of patients have experienced dyspnoea,
- Age, educational level, working status, financial status, history of hospitalization in the last year, non-COPD chronic illness, getting support for care affect sleep quality,
- Gender, marital status, family structure, social security status, smoking history variables do not affect the sleep quality of the patients,
- There is a strong relationship between dyspnoea severity and sleep quality of patients.

## Collaborations

We certify that all individuals who qualify as authors have been listed and each has participated in the conception and design of this work, collection of data, writing of the paper, and approval of the submission of this version.
